# Supplementary material for: Exercise modulation of the alternative splicing landscape in human tissues
Source: bioRxiv. 2026 Mar 4:2026.03.03.704460. Preprint. [Version 1] doi: 10.64898/2026.03.03.704460 (PMC13001507; doi:10.64898/2026.03.03.704460)
Supplement: Supplement 1 — Figure S1. Schematic illustrating the calculation of PSI values from RNA-seq reads. Top, rMATS uses the number of junction reads to quantify the transcripts including the alternative regions and the transcripts excluding the alternative regions, and calculate the PSI values based on these quantifications. Bottom, SUPPA uses all reads and quantifies distinct transcripts by pseudo-alignment of reads to annotations, and calculated PSI values based on the TPM values of quantified transcripts. Figure S2. Overlap between SKM DAS genes across time points within each exercise modality. Figure S3. A, Bar graph showing the proportions of adipose tissue genes associated with DAS events (“DAS genes”) that correspond to either non differentially expressed genes (DAS only) or differentially expressed genes (DAS-DEGs), and their distribution by AS event type, time point, and exercise modality. B, Same as A for blood genes. Related to Fig. 2B. Figure S4. Bar graph showing the proportions of adipose tissue DAS genes associated with a protein-coding sequence (“CDS”) change, a likely CDS change, or no CDS change. Provided to the right of the bar graph is a schematic describing each of the three types of changes resulting from a detected AS event. Related to Fig. 2D. Figure S5. Pathway enrichment analysis on the SKM DAS genes identified at P24H, with the DAS genes separated into 3 groups: DAS genes that are common to EE and RE, DAS genes that are EE-specific, and DAS genes that are RE-specific. Related to Fig. 3B. Figure S6. Spliceosome-related genes that were differentially expressed at P15M and at P24H are highlighted in the KEGG Spliceosome Reference pathway (https://www.kegg.jp/pathway/map=map03040&keyword=spliceosome). Each spliceosome-related gene is symbolized by a box. Regulation by EE is represented on the half left of a gene box, while regulation by RE is on the half right. In the scale bar, red indicates the highest level of expression. Related to Fig. 3C. Figure S7. Weighte [file media-1.pdf]

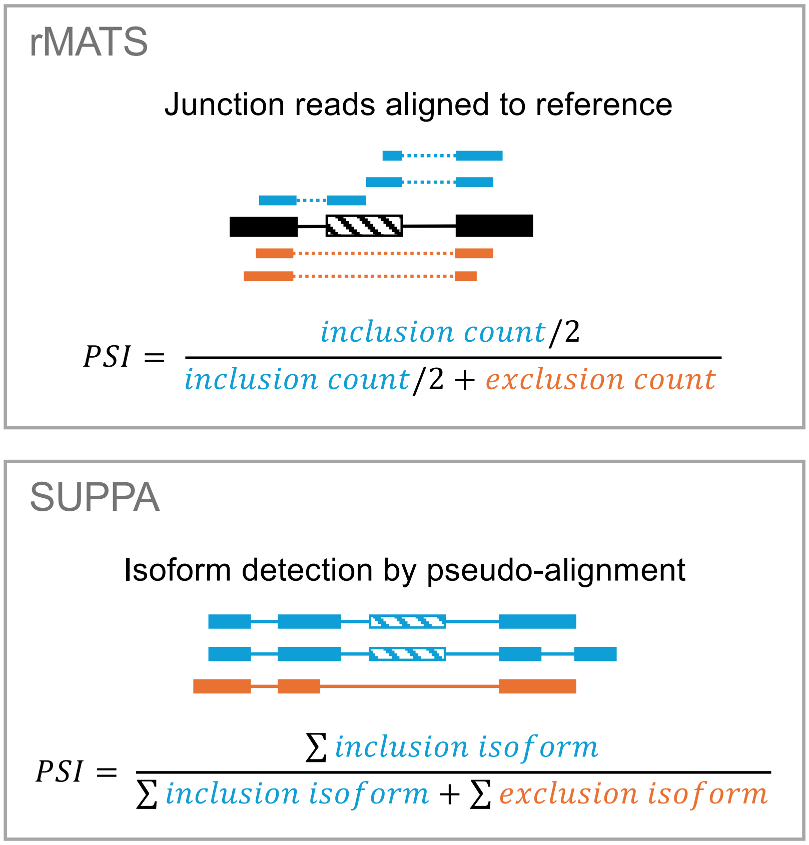

Supplementary Figure 1

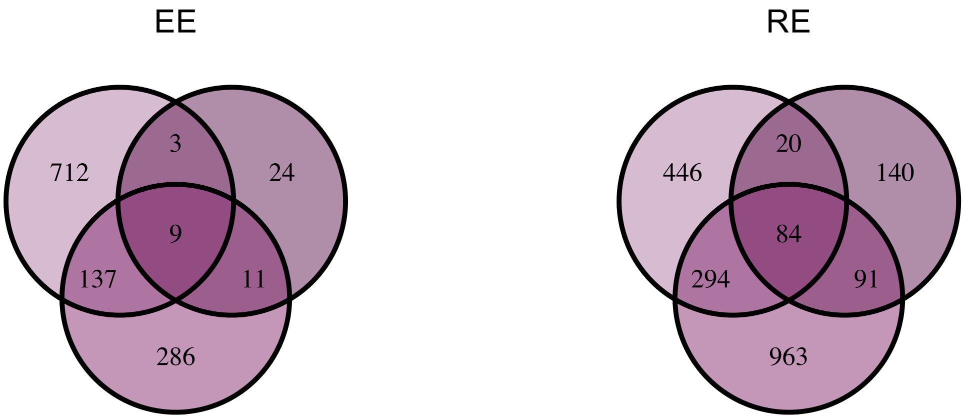

Supplementary Figure 2

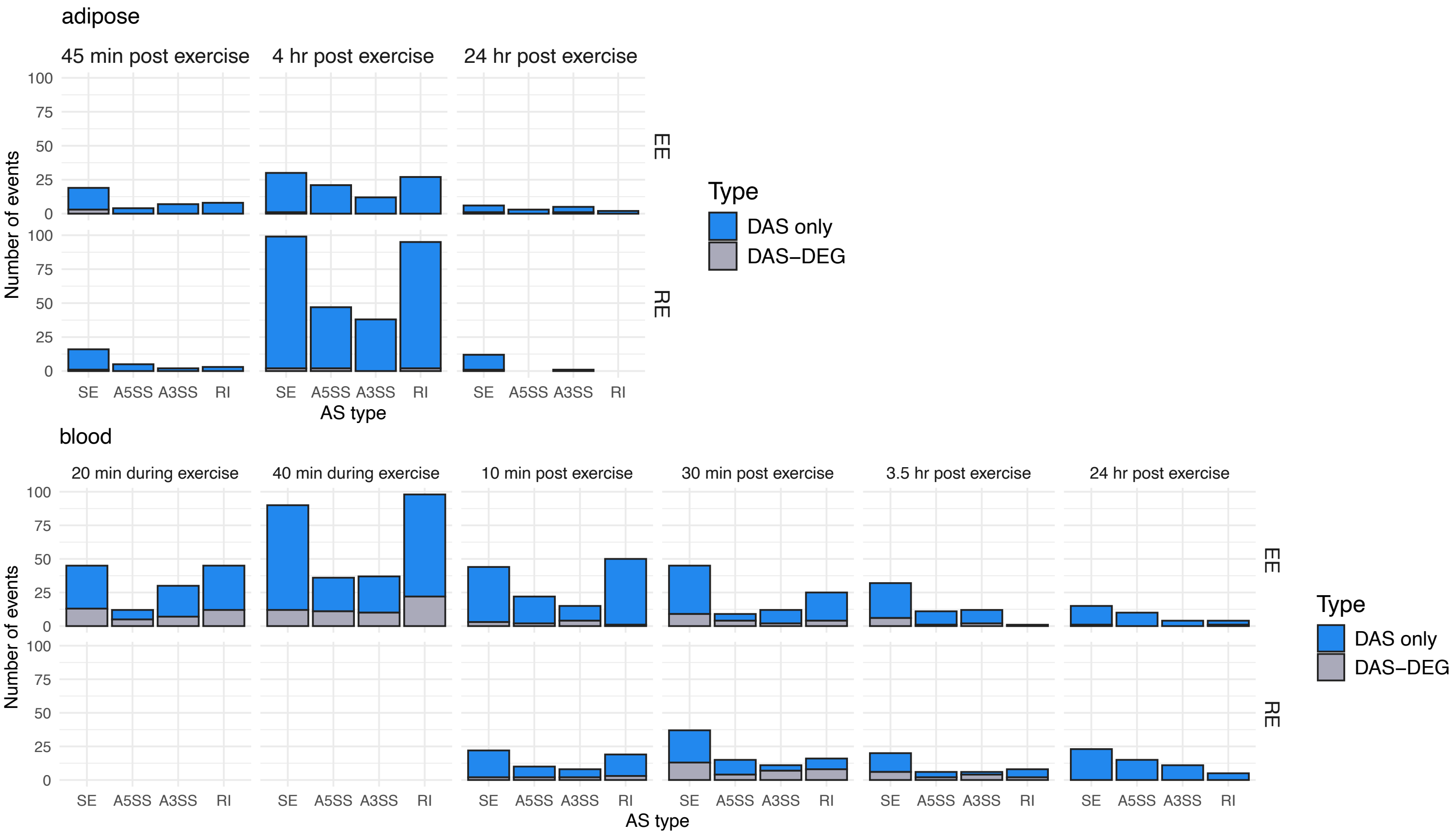

Supplementary Figure 3

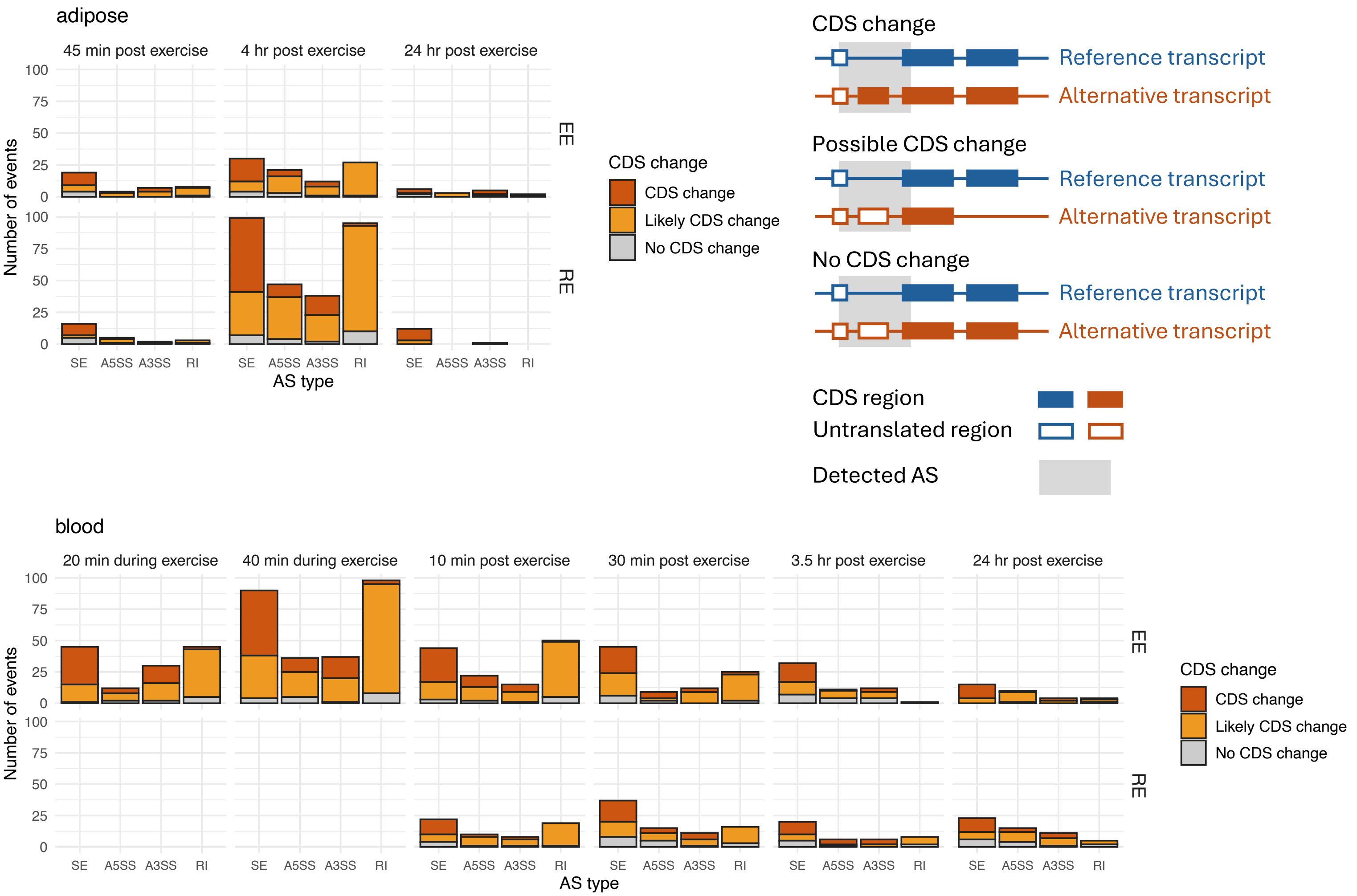

Supplementary Figure 4

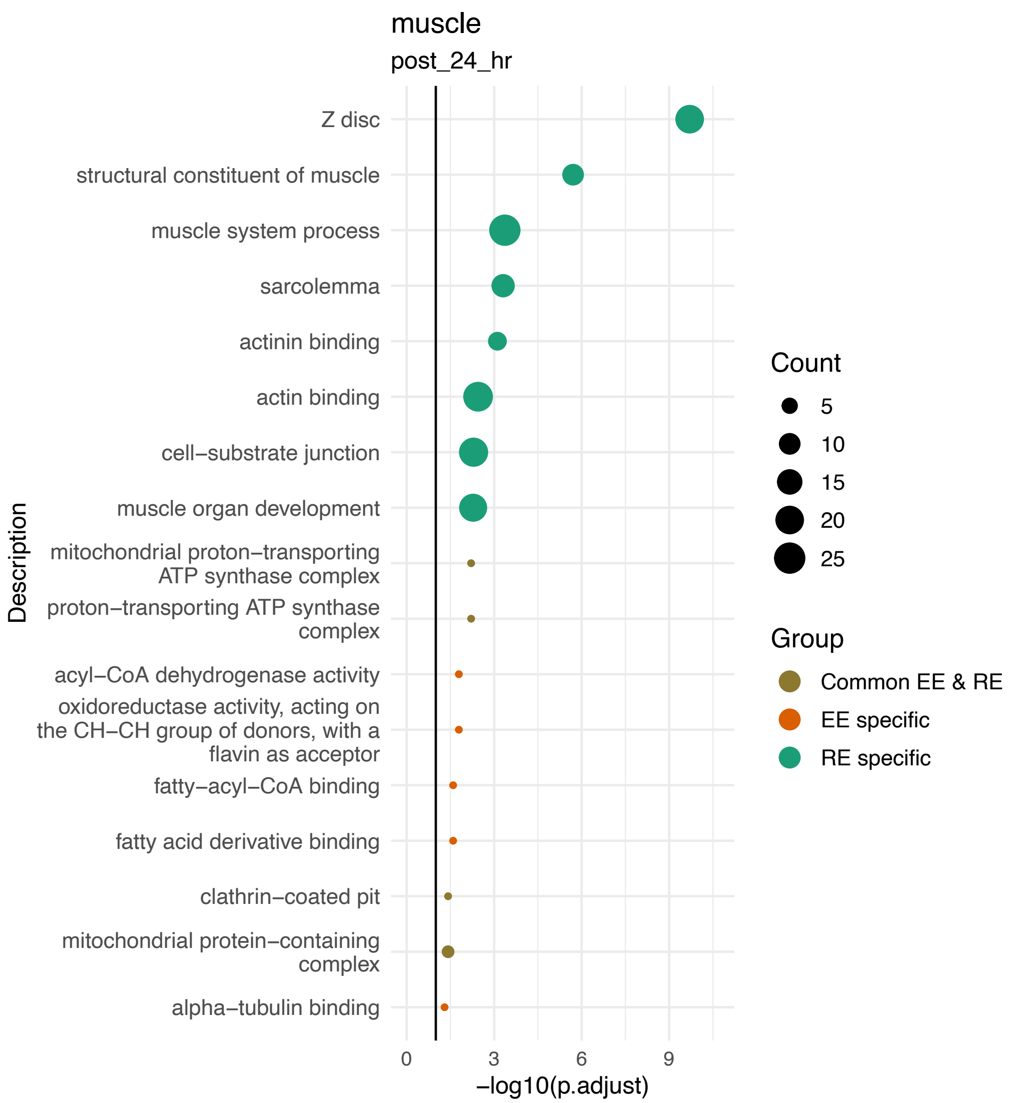

Supplementary Figure 5

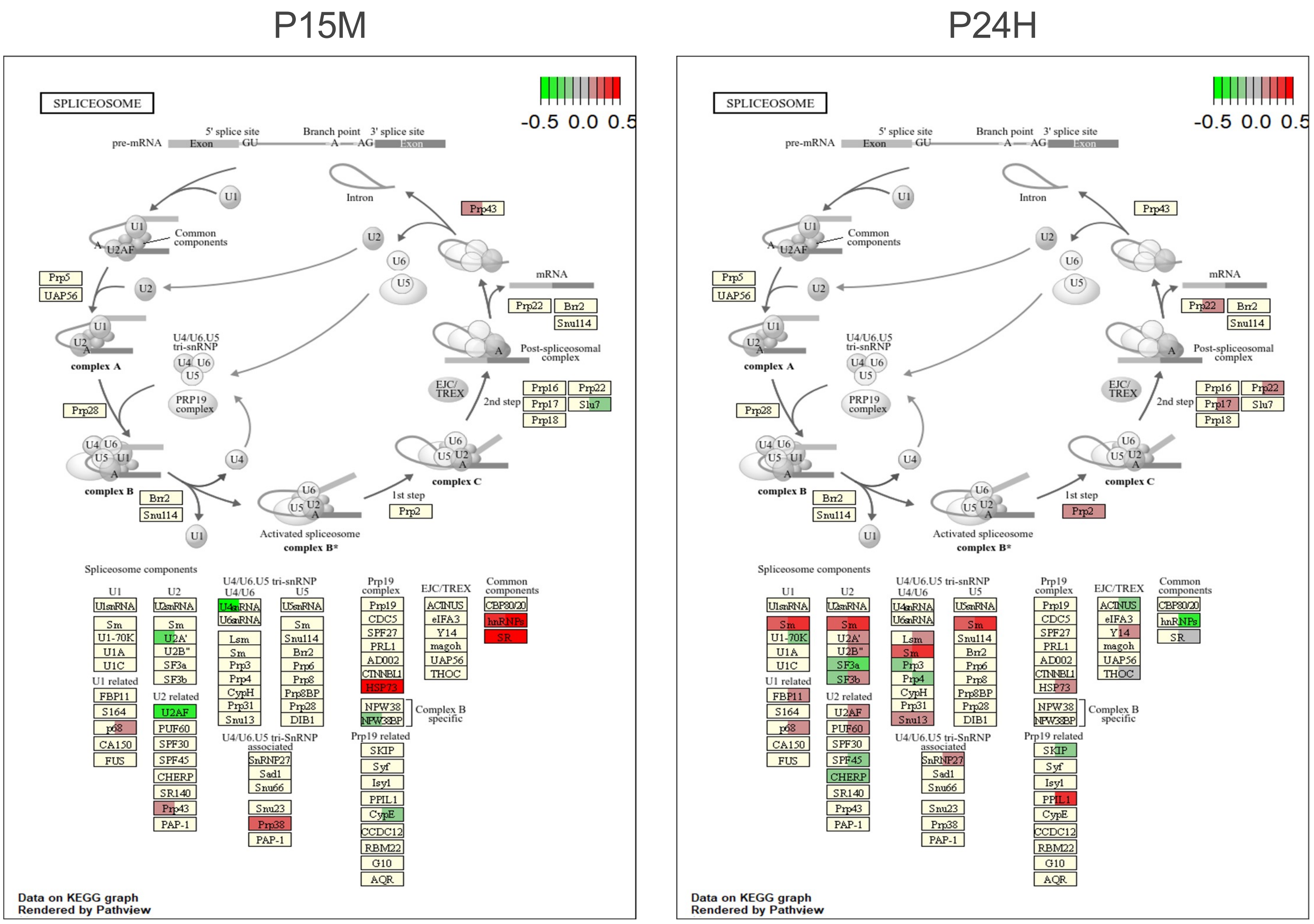

Supplementary Figure 6

MUSCLE

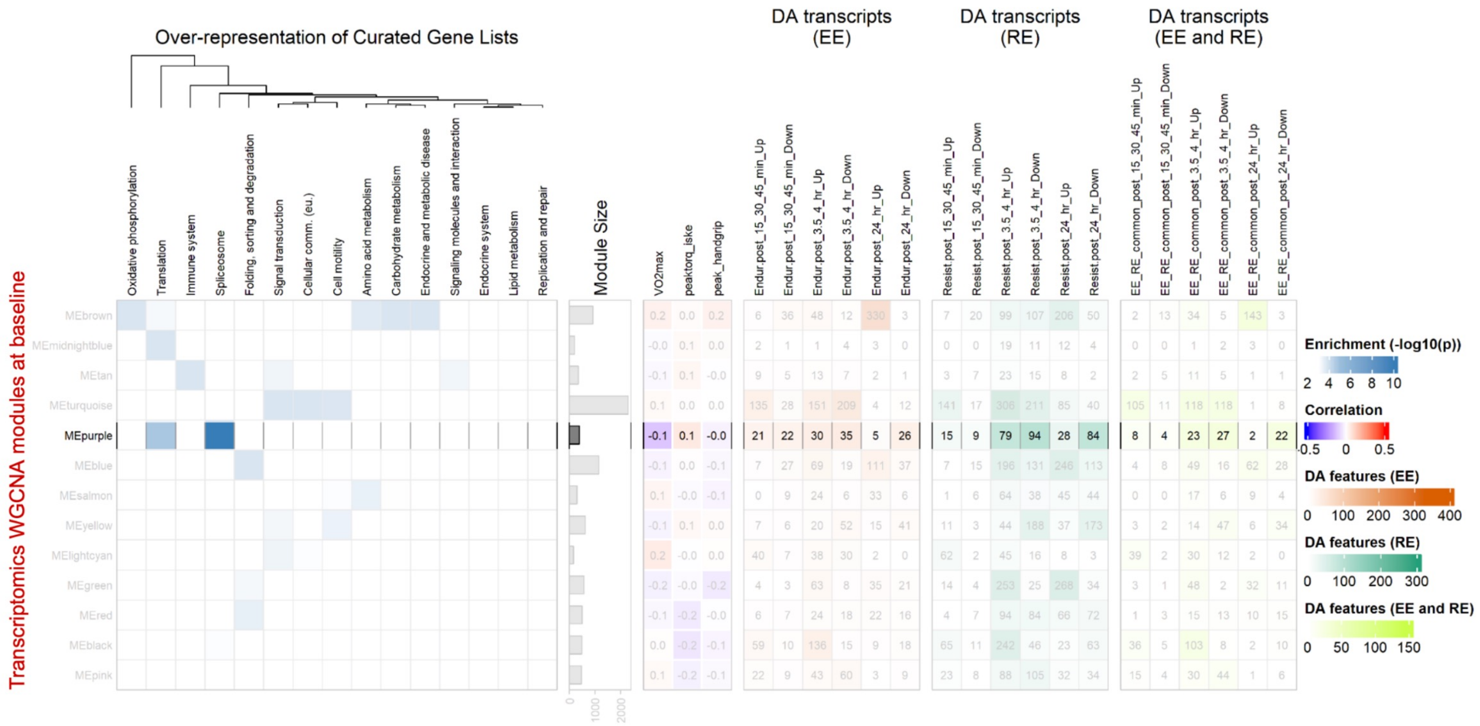

Supplementary Figure 7

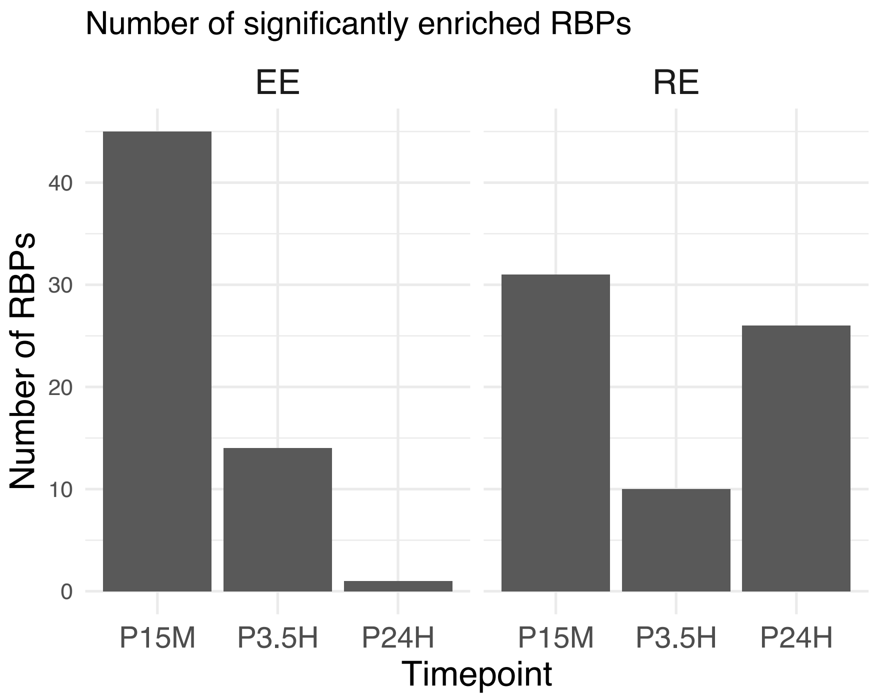

Supplementary Figure 8

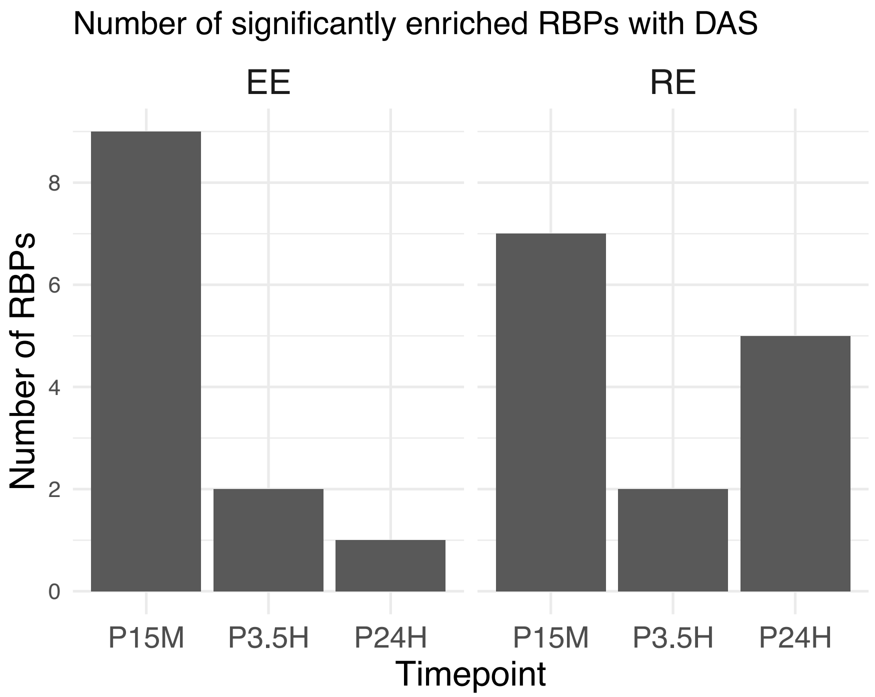

Supplementary Figure 9
